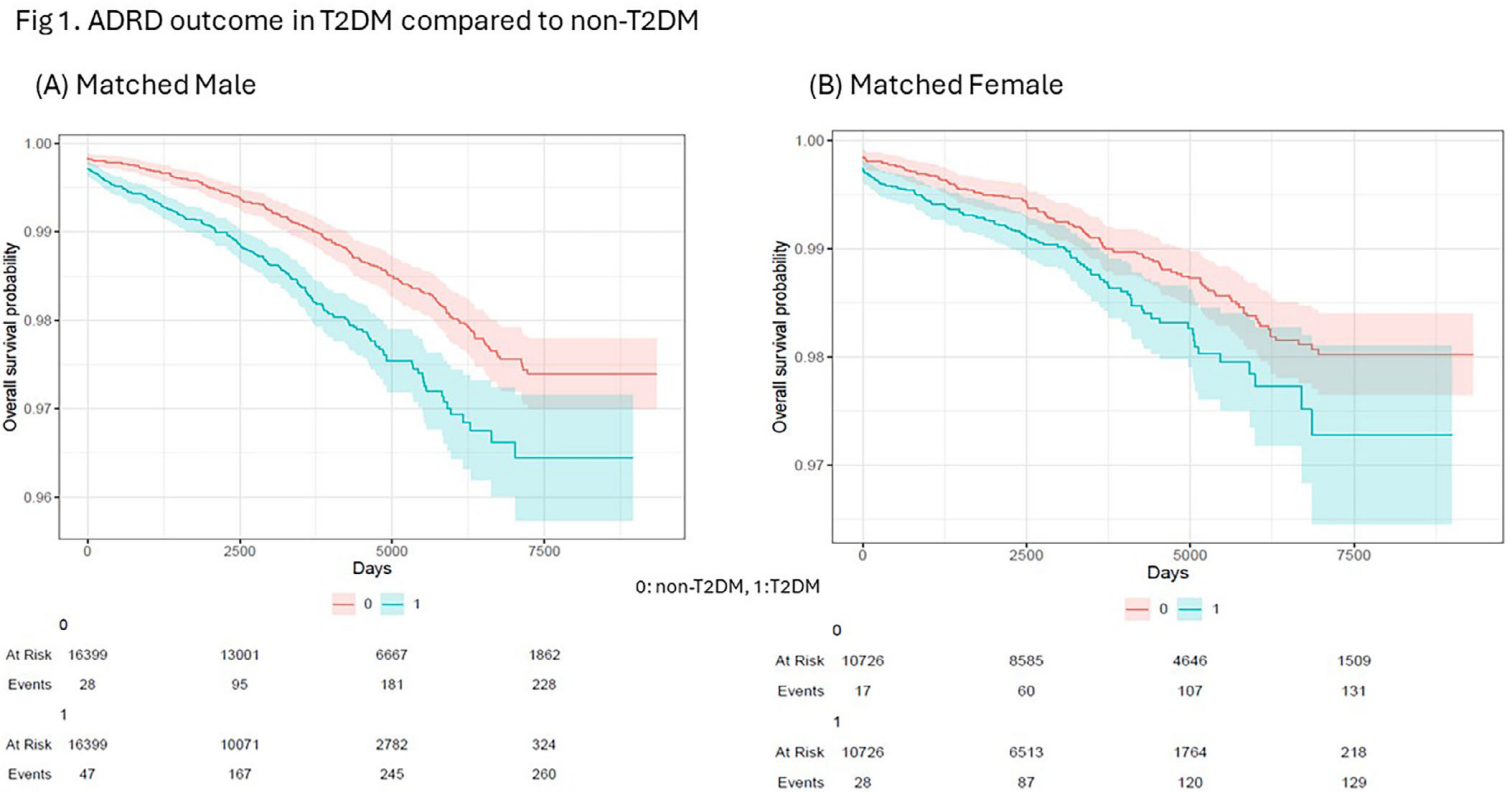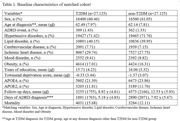# Late‐onset Alzheimer’s disease and related dementia in Type 2 Diabetes: 20‐year Longitudinal matched cohort study

**DOI:** 10.1002/alz.090794

**Published:** 2025-01-09

**Authors:** Sohyun Jeong, Alvaro Pascual‐Leone, Yi‐Hsiang Hsu

**Affiliations:** ^1^ Hebrew SeniorLife, Boston, MA USA; ^2^ Beth Israel Deaconess Medical Center, Harvard Medical School, Boston, MA USA; ^3^ Deanna and Sidney Wolk Center for Memory Health at Hebrew SeniorLife, Boston, MA USA; ^4^ Department of Neurology, Harvard Medical School, Boston, MA USA; ^5^ Broad Institute of MIT and Harvard, Cambridge, MA USA

## Abstract

**Background:**

Multiple studies report that patients with diabetes have an increased risk of Alzheimer’s disease and related dementia (ADRD). However, there are limited number of studies with a long‐term follow‐up and accounting for the strong genetic risk factor of *APOE* genotypes.

**Method:**

We used UK Biobank First occurrences (FO) datasets (Category 1712) in Health‐related outcomes data. Among the 406,049 eligible individuals, 27,125 T2DM cases were 1:1 matched to non‐T2DM by age at first diagnosis, sex, cerebrovascular disease, ischemic heart disease, hypertensive disorders, lipid disorders, obesity, and mood disorders. We applied the Cox proportional hazard model and Fine and Gray competing risk model with mortality as a competing risk. These models were adjusted for years of education, Townsend deprivation score, *APOE4* and *APOE2* genotypes to assess the ADRD outcomes. Additionally, for subgroup analysis by sex, *APOE4* and *APOE2* genotypes, we conducted matching within the subgroups and applied the same statistical models to assess ADRD risk in T2DM. We performed additional matching with T2DM polygenic risk score (PRS) and evaluated the non‐genetic T2DM effects toward ADRD risk.

**Result:**

The mean age at diagnosis was around 62 years old and female constituted 61% (Table 1). The ADRD risk was significantly increased in T2DM (HR:1.46, 95% CI: 1.23‐1.73). The Fine and Gray competing risk model presented 1.32 times increased risk in T2DM (95% CI: 1.12‐1.56). In the sex‐stratified analysis, a significantly increased risk was observed in males (HR:1.58, 95% CI:1.28‐1.95) but not in females with T2DM (HR:1.28, 95% CI:0.97‐1.69) (Fig. 1). In *APOE* stratified analysis, T2DM was not associated with ADRD risk in both *APOE4* and *APOE2* subgroups. T2DM PRS was negatively correlated with ADRD risk, but in T2DM PRS matched cohort, T2DM significantly increased ADRD (HR:1.52, 95% CI: 1.29‐1.80). Both male (HR: 1.62, 95% CI:1.31‐ 2.00) and female (HR:1.35, 95% CI:1.01‐1.79) subgroups presented elevated risks.

**Conclusion:**

We demonstrated a 1.3‐1.5 times increased risk of ADRD in T2DM. This finding was confirmed in PRS matched cohort, suggesting non‐genetic T2DM effects could contribute to ADRD risk.